# Weekly ILI patient ratio change prediction using news articles with support vector machine

**DOI:** 10.1186/s12859-019-2894-2

**Published:** 2019-05-20

**Authors:** Juhyeon Kim, Insung Ahn

**Affiliations:** 1 Department of data-centric problem solving research, Korea Institute of Science and Technology Information, Yuseong-gu, Daejeon, Korea; 2 Center for Convergent Research of Emerging Virus Infection, Korea Research Institute of Chemical Technology, Yuseong-gu, Daejeon, Korea

**Keywords:** Epidemics, Influenza, Machine learning, News article data, Support vector machine

## Abstract

**Background:**

Influenza continues to pose a serious threat to human health worldwide. For this reason, detecting influenza infection patterns is critical. However, as the epidemic spread of influenza occurs sporadically and rapidly, it is not easy to estimate the future variance of influenza virus infection. Furthermore, accumulating influenza related data is not easy, because the type of data that is associated with influenza is very limited. For these reasons, identifying useful data and building a prediction model with these data are necessary steps toward predicting if the number of patients will increase or decrease. On the Internet, numerous press releases are published every day that reflect currently pending issues.

**Results:**

In this research, we collected Internet articles related to infectious diseases from the Centre for Health Protection (CHP), which is maintained the by Hong Kong Department of Health, to see if news text data could be used to predict the spread of influenza. In total, 7769 articles related to infectious diseases published from 2004 January to 2018 January were collected. We evaluated the predictive ability of article text data from the period of 2013–2018 for each of the weekly time horizons. The support vector machine (SVM) model was used for prediction in order to examine the use of information embedded in the web articles and detect the pattern of influenza spread variance. The prediction result using news text data with SVM exhibited a mean accuracy of 86.7 % on predicting whether weekly ILI patient ratio would increase or decrease, and a root mean square error of 0.611 on estimating the weekly ILI patient ratio.

**Conclusions:**

In order to remedy the problems of conventional data, using news articles can be a suitable choice, because they can help estimate if ILI patient ratio will increase or decrease as well as how many patients will be affected, as shown in the result of research. Thus, advancements in research on using news articles for influenza prediction should continue to be pursed, as the result showed acceptable performance as compared to existing influenza prediction researches.

## Background

As Internet service has come into extensive use worldwide, it has enabled people to access fresh information faster and easier than ever before. For example, news articles can be viewed easily and quickly over the Internet, which was not the case when they were only available in newspapers. In the past, it was necessary to either receive a newspaper delivered at dawn or buy one from a kiosk to know what happened the previous day or recently; however, it is now possible to find this information through the Internet in real time. This has also allowed for real-time updates of infectious disease-related articles on the web being made available to people. Internet articles can be accessed indefinitely as long as the database that stores the article data does not disappear, and users can find, view, and use the data they want at any time. Therefore, since news is spread over the Internet, news articles can be collected and used as data over the Internet. As disease has a deadly effect on humans, many people are interested in this issue, and articles related to disease are quickly updated. The latest Internet-based disease reporting system also potentially provides a vast amount of data that can be incorporated into epidemiological models to examine disease distribution and transmission [1]. Influenza is one of the most infectious diseases affecting people all over the world, with related stories appearing globally almost every day.

Using machine learning techniques to predict the spread of influenza requires collecting data related to the spread of influenza. However, it is not easy to accumulate data for research, because only a few different types of data exist that are related to the spread of influenza to use as variables to predict the spread of influenza; further, since this data is often relevant to patients, it is necessary to collect data from patients and agencies with confidentiality agreements. Furthermore, even if these types of data are collectable, it is almost impossible to immediately obtain the latest data in a usable form. Study [2] used meteorological data for real-time influenza forecast while [3, 4] used ILI data from the CDC for real-time influenza forecasting. These studies showed acceptable performances, but they only considered influenza in the United States. Such meteorological and/or clinical data are easily collectable because the relevant systems are well constructed in the United States, while there are many obstacles to collecting data from many other countries. Thus, estimating influenza spread with the newest relevant data is challenging. Typically, for data on the number of influenza patients, there exists a delay of 2 to 4 weeks in reporting and/or publishing the data, which means it takes more than 2 to 4 weeks to convert raw data to usable data; therefore, techniques that can supplement that time are urgently needed. Most of the information provided through the Internet is free to read or use, and such data are recorded, accumulated, and updated in real time worldwide. Thus, if data that can be used for influenza prediction can be discovered, they would prove very useful.

Several different types of data can be obtained through the Internet, such as social network service articles, real-time search word statistics, and personal blog articles; some preceding studies have used data collected from the Internet. Reference [5] attempted to predict the number of influenza patients for 1 week using Google Flu Trends statistic data and climatic data. Some researchers have suggested that Twitter data can be used to predict the number of influenza patients because there is a high correlation between Twitter data and influenza-like illness (ILI) occurrence frequency [6]. The results of this study indicated that the predictions involving the groups of people aged ((5–24, 25–49)) years old showed the best outcome, because people in these groups use Twitter the most frequently. Reference [7] also suggested using Twitter data for influenza prediction. The authors collected 3.6 million flu-related tweets from 2008 to 2010 tweeted by 0.9 million Twitter users, and suggested a system that can be used to estimate the number of influenza patients in real time, using a probabilistic graphical Bayesian approach based on the Markov Network model. Reference [8] used Twitter and personal blog data with SVM and random forest regression to estimate the number of influenza patients. Furthermore, Reference [9] collected Twitter data from 2013 to 2014 and grafted them onto geographic information science in order to predict the number of influenza patients. However, as social network service data, real-time search word statistics, and data produced by personal blogs are not official sources of information, and include users’ subjective tendencies along with the relevant information, the accuracy of the information obtained is lower than that from news articles.

In order to remedy the problems of conventional data, in this study, features related to influenza spread are extracted from news articles provided by the Internet, and these are used to detect the variance in the number of influenza patients. The articles used for this research are collected from the web page of the Hong Kong Department of Health (Centre for Health Protection), and the data of the number of ILI, which is called the ‘Percentage of visits for ILI, National Summary’, are obtained from FluView, which is supplied by the United States CDC. The ILI data includes the number of ILI patients from 1997 to today in the United States. There are several sources that provide information about outbreaks and/or worldwide infectious disease information. However, this model was only capable of collecting data from 2004 to the present at CHP, while only recent articles are available on Healthmap or Medisys.

The remainder of this paper is organized as follows. Section 2 explains the methods of data extraction and preprocessing the extracted data. Section 3 introduces the proposed prediction model as well as the machine learning models called word2vec and SVM. Section 4 details the performance measures along with the experimental settings and results. Finally, Section 5 presents our conclusions.

## Methods

### Data

The climatic data that are used as features for influenza prediction can often be easily collected from national weather centers. These meteorological data are available from individual weather stations at hourly or multi-hour resolutions. However, only the weather stations of a few countries or regions provide these data. Furthermore, most of them do not provide long-standing data, such as data lasting 10 years. Moreover, the density of data from many weather centers is not that high, because weather centers typically provide weekly and monthly average data. Thus, in general, obtaining long-standing and high-density meteorological data from many countries is not easy. Low-density data are not suitable for predicting the number of weekly influenza patients, as there are not enough past data. Furthermore, the lack of past data is disadvantageous when using machine learning methods, because machine learning models show better performance with more data to learn. As the estimation of the number of influenza patients is concerned with disease spread, clinical data can be used for research; however, clinical data include personal information, so confidentiality agreements are required to collect clinical data, and sufficient time to obtain these confidentiality agreements is required as well. Even though access to clinical data can be granted through de-identifying data, and even though confidentiality agreements can be obtained in advance to cover both retrospective and future data, as there are thousands of different hospital organizations, it takes substantial amounts of time to merge the data from different organizations while using nationwide scaled clinical data. For these reasons, clinical data are not suitable for predicting the number of influenza patients in real time, which requires the newest data available, as it is difficult to gain such data and almost impossible to use the latest available data. Recently days, some studies have used data from Internet sources, such as social network services or personal blogs; however, these kinds of data may include the personal opinions of the data constructor or junk data such as spam mails. On the other hand, news articles can be collected through the Internet easily, and the Internet is updated in real time worldwide. Furthermore, most news articles are free to use. Some organizations such as Healthmap, Medisys, and the Centre for Health Protection publish news or reports about all kinds of international infectious disease, particularly about disease outbreaks and/or notifications. As the number of reports about a disease outbreak can represents the seriousness of that specific disease through the world, these articles are used as sources of variables for predicting the number of influenza patients [10]. However, as mentioned earlier, Healthmap and Medisys serves only the latest articles while Centre for Health Protection serves articles from 2004 to the present. Thus, in this research, in order to predict the number of influenza patients in the United States, we used news article data, which is easier to collect than traditional data for influenza prediction, such as climatic or clinical data. Moreover, as patterns of influenza emerge with correlates between countries, every article was used, regardless of which country it was describing [11]. The news article data we used were collected from the CHP web site, while the number of influenza patients in the United States was obtained from FluView, which is supplied by the United States CDC.

### News data

In total, 7791 news articles, which are composed of 93,326 words (3733 different words) and were generated from 2004.08 to 2018.02, were obtained from the CHP web page (https://www.chp.gov.hk/); the article links can be found at the webpage of CHP – Media Room – Press Releases Board. Each article includes the subject, updated date, and content of the article. Data accumulated from personal blogs and SNSs, such as Twitter, require additional filtering, because the data may include spam advertisements; however, news articles collected from the CHP do not require filtering. News articles from the CHP are open, meaning that anyone can access and use them for free.

**Fig. 1:**
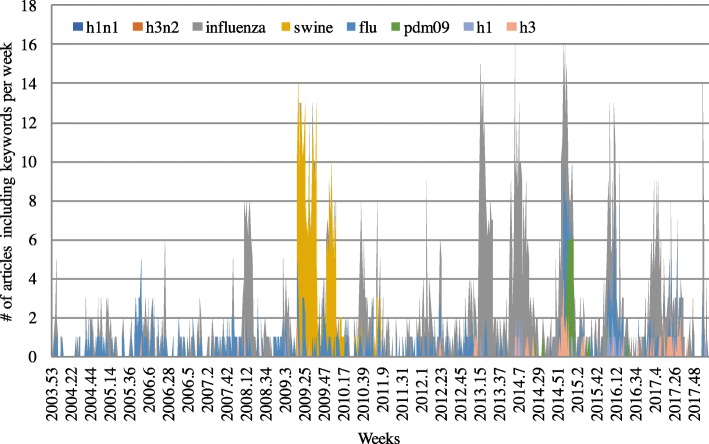
Total weekly counts of news articles including the eight keywords of ‘influenza’, ‘h1n1’, ‘h3n2’, ‘swine’, ‘flu’, ‘pdm09’, ‘h1’, and ‘h3’ from 2004.01–2018.02

CHP only supplies news articles related to infectious diseases, and some of them are closely connected to influenza. In order to estimate the weekly variance of the number of influenza patients, we used the weekly counts of the articles that include influenza related keywords as input variables. Thus, it was necessary to extract keywords that were highly related to influenza. The method for extracting keywords is called word2vec, and it is explained in Section 3. After vectorizing the words in articles using word2vec, the top 15 words most similar to influenza were extracted, using the multiplicative combination objective proposed by Omer Levy and Yoav Goldberg in [12 Linguistic Regularities in Sparse and Explicit Word Representations]. However, avian influenza-related keywords are also pulled out, because words similar to influenza are extracted. Thus, keywords related to avian influenza such as ‘h7n9’ are terminated. In addition, general keywords such as ‘human’ are terminated because these keywords cannot represent the characteristics of influenza. The result of using word2vec gave us seven different keywords ‘H1N1’, ‘H3N2’, ‘swine’, ‘flu’, ‘PDM09’, ‘H1’, and ‘H3’, which are strongly connected to influenza when these words are vectorized. The weekly counts of news articles that include each of these seven highly influenza-related keywords as well as the keyword ‘influenza’ are calculated as shown in Fig. 1. The weekly occurrence frequency of news articles can be expressed as Eq. (1):

1 $$ X=\left\{{x}_1,{x}_2,\dots, {x}_t\right\} $$

where *t* means the order of the weeks and *x*_*t*_ means the counted number at week *t*. For example, data for ‘H1N1’ can be expressed as ${X}^{h1n1}=\left\{{X}_1^{\# of\ h1n1},{X}_2^{\# of\ h1n1},\dots, {X}_t^{\# of\ h1n1}\right\}$. As the counts of weekly keyword occurrence at news articles are time series data, technical indicators (TI) are applied to these data in order to analyze the data effectively. Table 1 shows the TIs used in this research. TIs are often used in predictive experiments with time series data, because they can reduce the noise on the vibrations of time series data [12, 13].

**Table 1:** Explanation of six different TIs

Technical Indicator	Meaning
${MA}_p\left({X}_t\right)=\frac{1}{p}\left({x}_t\right)+\frac{p-1}{p}{MA}_p\left({X}_{t-1}\right)$	*p*-moving average (exponential smoothing)
${BIAS}_p\left({X}_t\right)=\frac{x_t-{MA}_p\left({X}_t\right)}{MA_p\left({X}_t\right)}$	Change rate of *x*_*t*_ relative to *MA*_*p*_(*X*_*t*_)
${ROC}_p\left({X}_t\right)=\frac{x_t-{x}_{t-p}}{x_t}$	Relative rate of change for *x*_*t*_ between *p* consecutive time points
${K}_t^p=\frac{x_t-{\mathit{\operatorname{Min}}}_{i=t-p-1}^t\left({x}_i\right)}{{\mathit{\operatorname{Max}}}_{i=t-p-1}^t\left({x}_i\right)-{\mathit{\operatorname{Min}}}_{i=t-p-t}^t\left({x}_i\right)}$	Standardization of *x*_*t*_
${D}_t^p={MA}_3\left({K}_t^p\right)$	3-Moving Average of ${K}_t^p$
$$ {RSI}_t^p=100-\frac{100}{1+\frac{\sum_{i=t-p-1}^t\left\{\begin{array}{c} if\ {x}_i-{x}_{i-1}>0,\mid {x}_i-{x}_{i-1}\mid \\ {} if\ {x}_i-{x}_{i-1}<0,0\end{array}\right.}{\sum_{i=t-p-1}^t\left\{\begin{array}{c} if\ {x}_i-{x}_{i-1}<0,\mid {x}_i-{x}_{i-1}\mid \\ {} if\ {x}_i-{x}_{i-1}>0,0\end{array}\right.}} $$	Relative strength index

Seasonal influenza typically occurs between November and March in the Northern Hemisphere, and between April and September in the Southern Hemisphere. As CHP is located in the Northern Hemisphere, most news articles published from CHP involve countries located in the Northern Hemisphere. In practice, out of the 7791 news articles collected, only about 5.25% of them involve countries located in the Southern Hemisphere. Thus, we constructed two different article groups, where the first one is constructed with every news article collected while the second one is constructed with news articles excluding the articles about countries in the Southern Hemisphere so as to compare which data can predict the Weekly ILI patient ratio changes in United States.

### Epidemiological surveillance data

**Fig. 2:**
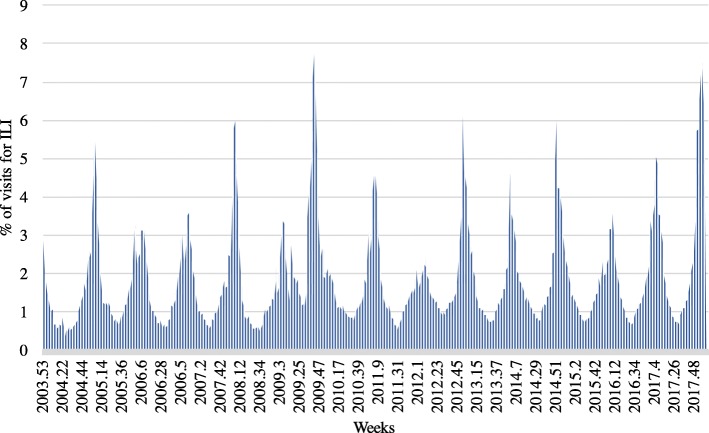
Percentage of hospital visitors due to ILI in the United States as provided by the CDC

In order to predict the trend of the influenza population, a collection of actual influenza cases is required, and these data are typically generated by doctors or researchers at medical institutions. This research uses influenza surveillance data from the United States CDC, which provides online statistics regarding flu patients on a national basis every week. The hospital visit rate data of patients due to ILI per week are used. Figure 2 shows the data collected, and the data used in the study are published data that can be accessed and used by anyone.

The experiment first used the data collected to predict whether the number of patients would increase or decrease over the previous week. Thus, every week had to be labeled using the data of the rate of visits to hospitals for the ILI data. If the number of patients visiting the hospital had increased compared to the previous week, then the given label became ‘+ 1’, and if the number had decreased, the given label became ‘-1’, which can be expressed as Eq. (2):

2 $$ {y}_t=\mathit{\operatorname{sign}}\left({x}_t^{\# of\ infectee}-{x}_{t-1}^{\# of\ infectee}\right) $$

For example, if the number of patients at week *t* was less than the number of cases at *t* − 1, then week *t* was given ‘-1’, and in the opposite case, the label ‘+ 1’ was assigned. As there were no consecutive cases having the same rate of patients, every week could be labeled as either ‘+ 1’ or ‘-1’.

### Word2Vec

Natural Language Processing (NLP) is a technique that allows a computer to recognize and analyze human language. In order to enable the computer to recognize a certain word, the word should be expressed as a numerical value, which was a challenging problem in the past. Word vectorization was proposed to solve this problem. If words can be vectorized, then it is possible to do such things as calculate the similarity between words, or to find the average place of several words. Every word embedding-related learning process is based on the assumption of the distributional hypothesis, which means that words with a similar distribution have similar meanings. A similar distribution means that words appear in a similar context; for example, if a paragraph frequently contains certain words, then we can infer that these words may have similar meanings. Although it is not easy to identify these relationships with a small number of learning data, learning a great deal of text data will facilitate the understanding of the relationship between these words. Word2vec is a natural language processing technique that is a continuous word embedding learning model composed by Google engineers including Mikolov in 2013 [14, 15]. They named their method as ‘Word2vec’, and this method allowed for the vectorization of words in sentences or paragraphs. Word2vec is not that different from a neural network, which is a traditional machine learning method for word vectorization, but its processing speed is several times faster by greatly reducing the computation, and it has thus become the most widely-used word embedding method. In contrast to traditional methods, word2vec presents two different network models for learning: the Continuous Bag-of-Words (CBOW) and the Skip-gram model. In this experiment, the CBOW was used to extract keywords. The CBOW model uses a total of C words in input, C/2 before and after a given word, and creates a network to match a given word. Word2vec was applied to 7791 news articles composed of 93,326 words that were crawled from CHP.

### Support vector machine

The object of SVM is to identify an optimal decision boundary that is divided by maximizing the margin between the nearest samples of two different data groups [16]. SVM uses input-output pairs, such as *D = {(x*_*1*_*, y*_*1*_*), (x*_*2*_*, y*_*2*_*),* …, *(x*_*ℓ*_*, y*_*ℓ*_*)}*, where i = 1, …, ℓ for classification, and x ∈ X and y ∈ Y. ‘Y’ represents the classes and can be expressed as Y = {− 1, + 1}. Typically, in binary classification problems, the training data set is divided into two different groups by a hyperplane, which can be composed in a linear or non-linear form. In the linear classification cases, the optimal linear decision function that can precisely divide the training data is calculated [17]. If two different classes cannot be divided by the linear function because there noise data exist, users set an error tolerance and use linear classification. In this case, identifying the optimal hyperplane that maximizes the margin between two different data groups and minimizes misclassification is necessary. SVM finds the maximum margin between two different classes by using Eq. (3) [18]:

3 $$ {\displaystyle \begin{array}{c}\min \kern1em \Theta \left(\overrightarrow{\mathrm{w}},\upxi \right)=\frac{1}{2}{\overrightarrow{w}}^2+\complement \sum \limits_i^M{\upxi}_{\mathrm{i}},\\ {}s.t.\kern1.5em {y}_i\left(\overrightarrow{w}.\Phi \left(\overrightarrow{x_i}\right)+b\right)\ge 1-{\upxi}_{\mathrm{i},}\\ {}{\upxi}_{\mathrm{i}}\ge 0,i=1,\dots ..,M.\end{array}} $$

Parameter C in Eq. 3 is the penalty for misclassified data. The larger the value of C is, the fewer cases there will be of misclassification of the SVM model [17]. Parameter ξi is a non-negative slack variable that decides the limit of misclassification when misclassification cannot exist. If, because of the essential problem, data is divided by a non-linear hyper plane, mapping input features into a high-dimensional feature space that can be divided by a linear hyperplane may be an appropriate solution. Such mapping can be done by a kernel function. In this research, the RBF kernel $k\left({x}_1,{x}_2\right)={e}^{-\gamma {\left|{x}_1{x}_2\right|}^2}$, which is the most widely used, is applied [19]. The tradeoff parameter C and kernel width γ are set by the user, and these parameters are concerned with the performance of SVM.

### Analysis

**Fig. 3:**
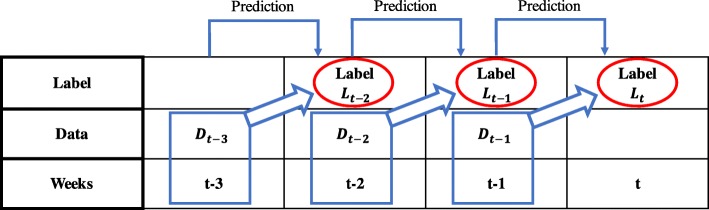
Use of data *D*_*t* − 1_ to predict label *L*_*t*_

Several techniques were used to extract the necessary data from the collected news article data and predict if the number of ILI patients will increase or decrease. First, while reproducing news article data as time series data, it was necessary to extract several keywords that were closely related to influenza, because more data leads to better performance of the prediction model. In order to determine which keywords were related to the keyword ‘influenza’, we used word2vec. Then, SVM, which is widely used for classification problems, was applied to the extracted data to predict if the number of influenza patients would increase or decrease at a specific week. In order to predict the future status, experiments were conducted as described in Fig. 3. For example, data *D*_*t* − 1_ was used to predict the label *L*_*t*_. This method allows for one-week ahead weekly ILI patient ratio change prediction.

**Fig. 4:**
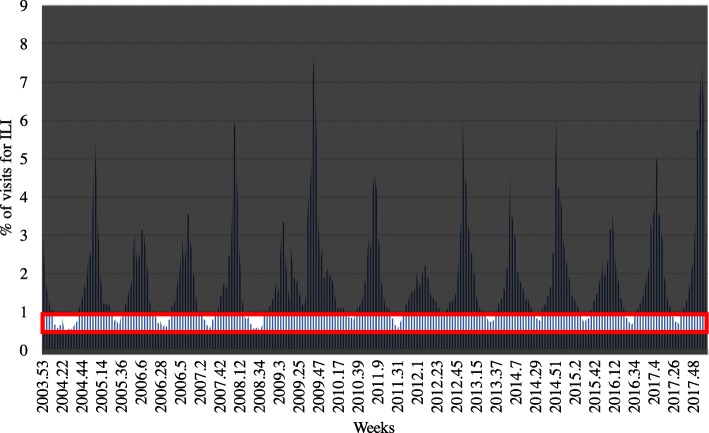
Proportion of hospital visitors from (1 to 1.5) % due to ILI

**Fig. 5:**
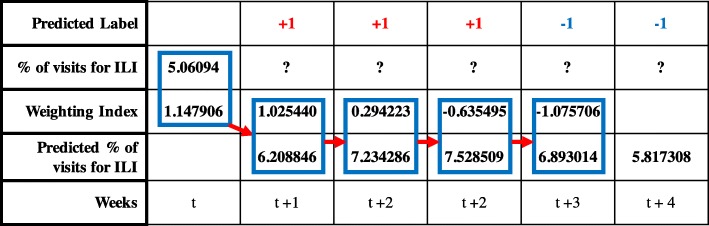
Method for measuring the number of flu patients using the weighting index

After predicting the fluctuation of the number of ILI patients, an assumption was made to define a weighting index and estimate the rate of visits to hospitals for ILI. Fig. 2 shows the ratio of hospital visitors due to the ILI; when the number of patients is at a certain level, if the number of patients at week *t* is *n*_*t*_, then *n*_*t*_ − *n*_*t* − 1_ can significantly decrease; by contrast, when it is above a certain level, *n*_*t*_ − *n*_*t* − 1_ can significantly increase. Therefore, we assumed that the change in the number of patients would be similar at a certain level. For example, when the ratio of hospital visitors due to the ILI is between (0.5–1.0) %, shown in the red box in Fig. 4, the average change of ratio when the patients increased is 0.089841 and the average change of ratio when the patients decreased is − 0.0076988, respectively. We assumed that the change in the ratio of hospital visitors due to the ILI would be exactly the same as 0.089841 or − 0.0076988 every week if it is in the ratio of (0.5–1.0) %. Thus, the change of ratio at 15 different levels (0–0.5, 0.5–1.0, 1.0–1.5, 1.5–2.0, 2.0–2.5, 2.5–3.0, 3.0–3.5, 3.5–4.0, 4.0–4.5, 4.5–5.0, 5.0–5.5, 5.5–6.0, 6.0–6.5, 6.5–7.0, 7.0-) when the number of patients increases and decreases are calculated and used as weights. Through the same method, the weighting index was created as shown in Table 2 by calculating the weight values for each interval by dividing the intervals by 0.5% of the patients. With the intended weighting index and the predicted results of variance of the number of influenza patients, the proportion of patients visiting the hospital due to ILI can be estimated. For example, as shown in Fig. 5, if the rate of visits to hospitals for ILI at week *t* is known and the fluctuations are predicted from week *t* + 1 to *t* + 4, then the rate at *t* + 4 can be estimated. As the rate at week *t* is located between (5–5.5) %, and if the number of patients will increase at week *t* + 1, as it is predicted to +1, then the rate at *t* + 1 will become 6.208846, which is the sum of 5.06094 and 1.147906. The rate at week *t* + 4 can be determined by repeating the method by applying the weighting index.

**Table 2:** Defined weighting index by rate of hospital visits for ILI. The columns with ‘Increasing’ in their name present values when the ILI patient ratio increases, while the columns with ‘Decreasing’ in their name present values when the ILI patient ratio decreases. Columns with ‘Weight’, ‘Max’, ‘Min’, and ‘Variance’ in their name present the weighting index, maximum variation between 2 weeks in the corresponding section, minimum variation between 2 weeks in the corresponding section, and the variance of the corresponding section, respectively

Increasing Position	Increasing Weight	Increasing Max	Increasing Min	Increasing Variance	Decreasing Weight	Decreasing Max	Decreasing Min	Decreasing Variance
7 –	0.29422	0.62043	0.09362	0.05626	−0.63550	− 0.27382	− 0.99717	0.67810
6.5–7	0.73653	0.82521	0.64785	0.01573	−1.07571	−0.52189	−1.46485	0.24263
6–6.5	1.02544	1.02544	1.02544	0.07129	−1.27125	−0.93999	−1.46226	0.08295
5.5–6	0.26870	0.58781	0.05424	0.04053	−0.92872	−0.32721	−1.72300	0.28525
5–5.5	1.14791	1.98829	0.24014	0.61766	−1.11697	−0.20106	−2.24922	0.44621
4.5–5	0.88616	1.18313	0.47218	0.06205	−0.60852	−0.08655	−1.28999	0.17022
4–4.5	0.68902	1.75528	0.04946	0.38890	−0.41227	−0.00352	− 0.79770	0.08223
3.5–4	1.00738	1.90345	0.29164	0.26339	−0.39826	−0.05063	− 0.90527	0.06080
3–3.5	0.50036	1.43056	0.00657	0.20447	−0.38381	−0.00493	−1.01177	0.07739
2.5–3	0.64434	1.60591	0.00712	0.18164	−0.35944	−0.03027	−1.01787	0.05589
2–2.5	0.34778	1.06664	0.06004	0.05790	−0.26849	−0.00097	− 0.82067	0.04081
1.5–2	0.25105	0.93668	0.00259	0.03375	−0.20100	−0.00405	− 0.80927	0.02859
1–1.5	0.13309	1.44692	0.00003	0.03668	−0.10936	−0.00031	−1.33997	0.02095
0.5–1	0.08984	0.91575	0.00043	0.01425	−0.07699	−0.00032	− 0.70360	0.01017
0–0.5	0.12861	0.24557	0.026765	0.00591	−0.19103	−0.02152	− 0.49434	0.04773

### Process summary

**Fig. 6:**
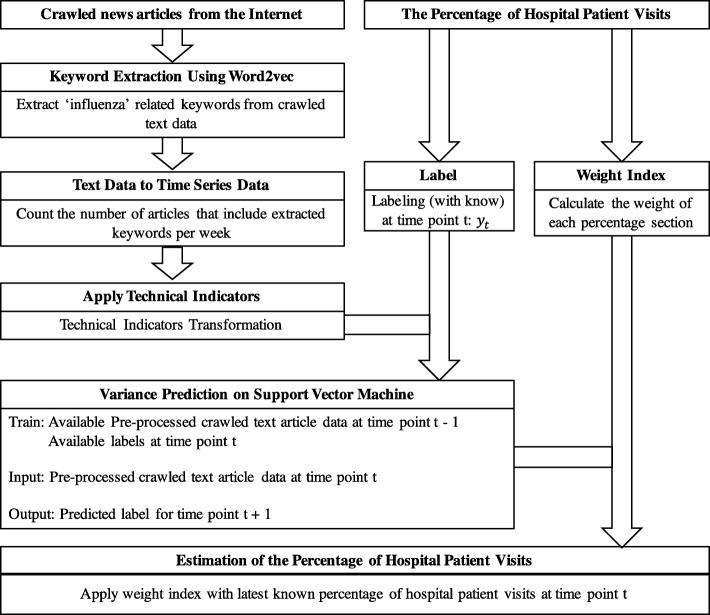
Summary of the proposed method

The method for estimating the number of influenza patients in this research can be summarized as follows: First, news articles related to infectious diseases are collected, then keywords that are highly connected with ‘influenza’ are extracted. After extracting the relevant keywords, time series data are generated by taking weekly counts of the number of news articles that include each keyword. Then, technical indicators are applied to the generated time series data so as to avoid noise. The rate of visits to hospitals for ILI data used as predictors is collected, and labels are made for each week using the predictor. Every label is composed of ‘+ 1’ or ‘-1’ using the proposed method. Then, the weighting index needs to be defined in order to estimate the exact rate of visits to hospitals for ILI. After preprocessing the collected raw data, SVM is applied to predict if the number of patients increases or decreases at a certain week. If data until time point *t* are collected, then the label until *t* and news article data until *t* − 1 would be used to train the model. Using the trained model, we predict whether patients will increase or decrease at time point *t* + 1 using the news article data at time point *t*. Following this prediction, we estimate the real value of the rate at *t* + 1, applying the weighting index. Figure 6 summarizes the proposed method. Several weeks are required to obtain the ILI patient ratio of the current week (the latest ILI patient ratio data provided by the CDC of the United States are data from 2 to 3 weeks prior to the current week). Therefore, *t* + 2 and *t* + 3 can be predicted using published article data with the trained model in the same way as *t* + 1.

## Results

**Fig. 7:**
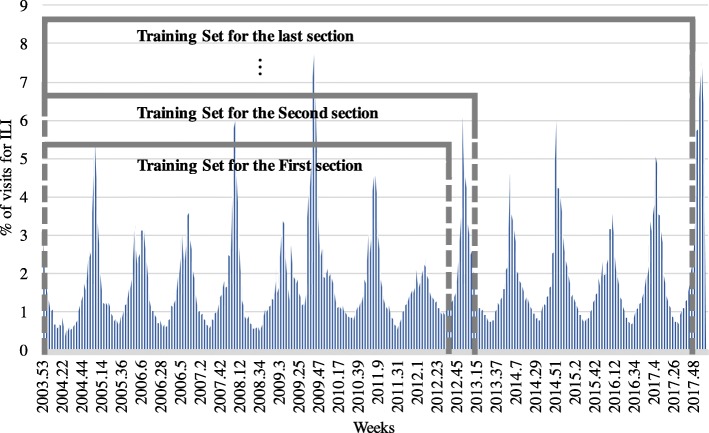
Change in the training set as the experiment progressed

The CHP news articles data and rate of visits for hospitals for ILI data collected for a total of 753 weeks from the 32nd week of 2003 to the 10th week of 2018 were used in this research. The data over the 240-week period from the 32nd week of 2013 to the 10th week of 2018 were used for the validation set. The 240 weeks are divided into 20 different groups of 12 weeks in order to see if it is available to predict the quarter of a year ahead, even if the ILI patient ratio data does not exist. Figure 7 shows that while the experiment progresses, the training set increases. For example, if the third section from the 5th week of 2014 to the 16th week of 2014 out of the 12 sections was to be predicted, then the data until the 4th week of 2014 would be used to train the model.

SVM was used to predict patient variation, and the RBF kernel, which showed the best performance, was applied. For parameter setting, the C and *γ* that gave the best prediction performance were identified from the combinations of {*γ*, *c*} ∈ {10^−9^, 10^−8^, 10^−7^, 10^−6^, 10^−5^, 10^−4^, 10^−3^, 10^−2^, 10^−1^, 1, 10, 10^3^} × {10^−2^, 10^−1^, 1, 10, 10^3^, 10^4^, 10^5^, 10^6^, 10^7^, 10^8^, 10^9^, 10^10^}. Grid search was performed for every section, and the best parameter combinations found through grid search were used for each prediction. The performance of the prediction of whether the number of ILI patients would increase or decrease was measured by accuracy, and the estimation of the rate of visits for hospitals for ILI was measured by root mean square error (RMSE). Accuracy represents how many correct answers have been made in the total cases, and can be presented as Eq. (4), where *T*_*p*_ is true positive, *T*_*n*_ is true negative, *F*_*p*_ is false positive, and *F*_*n*_ is false negative. RMSE is a commonly used measure for the difference between the estimated value and the value observed in the actual environment, and can be expressed as Eq. (5).

4 $$ Accuracy=\frac{T_p+{T}_n}{T_p+{T}_n+{F}_p+{F}_n} $$

5 $$ RMSE=\sqrt{\frac{\sum_{i=1}^n{\left({x}_{1,i}-{x}_{2,i}\right)}^2}{n}} $$

**Fig. 8:**
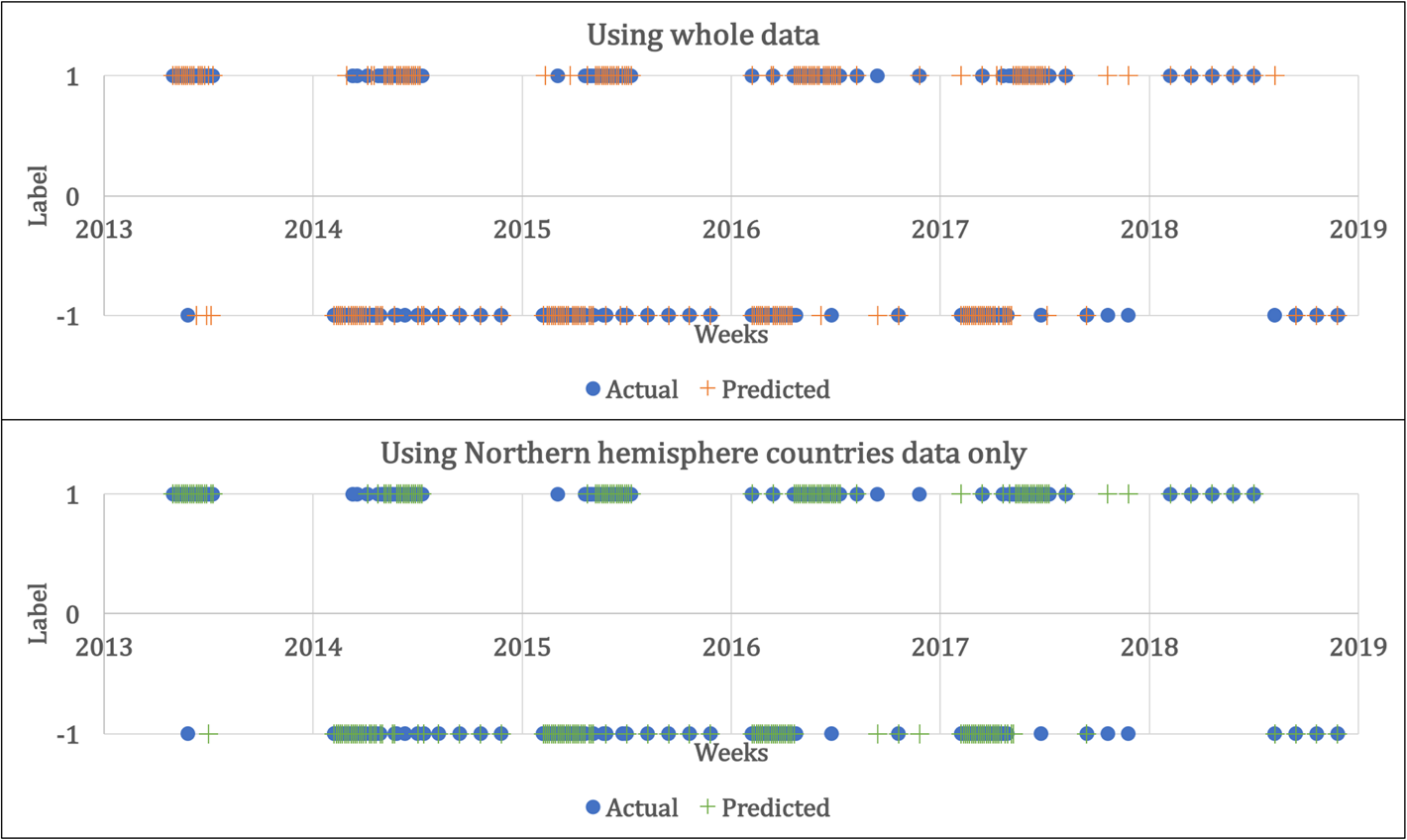
Results of prediction for whether the ILI patient ratio will increase or decrease from the 32nd week of 2013 to the 10th week of 2018

The results of the experiment are based on estimates of the expected accuracy of the predictions of whether a patient’s hospital visit rate due to ILI would increase or decrease as well as the actual value of rate of visits of hospitals for ILI. Figure 8 shows the prediction results for variations in patients from the 32nd week of 2013 to the 10th week of 2018, where, ‘1’ means the patient increased and ‘-1’ means the patient decreased. Blue dots represent the actual variances, and the orange and green crosses represent the predicted results using all of the collected data and data from Northern Hemisphere countries only, respectively. The only-orange/green cross or -blue dot in the graph is the part that the predictive model predicts differently than the actual value, and the model predicted 209 cases correctly out of 240 cases when using all collected data, with 31 wrong, while the model predicted 210 cases correctly out of 240 cases when using only the data from Northern Hemisphere countries, with 30 wrong. Table 3 shows the confusion matrix.

**Table 3:** Confusion matrix for the experimental results of using all data and using data from Northern Hemisphere countries only

Predicted	Using all data			Using Northern Hemisphere countries data only		
Actual	Positive	Negative	Total	Positive	Negative	Total
Positive	106	15	121	105	16	121
Negative	16	103	119	14	105	119
Total	122	118	240	119	121	240

**Fig. 9:**
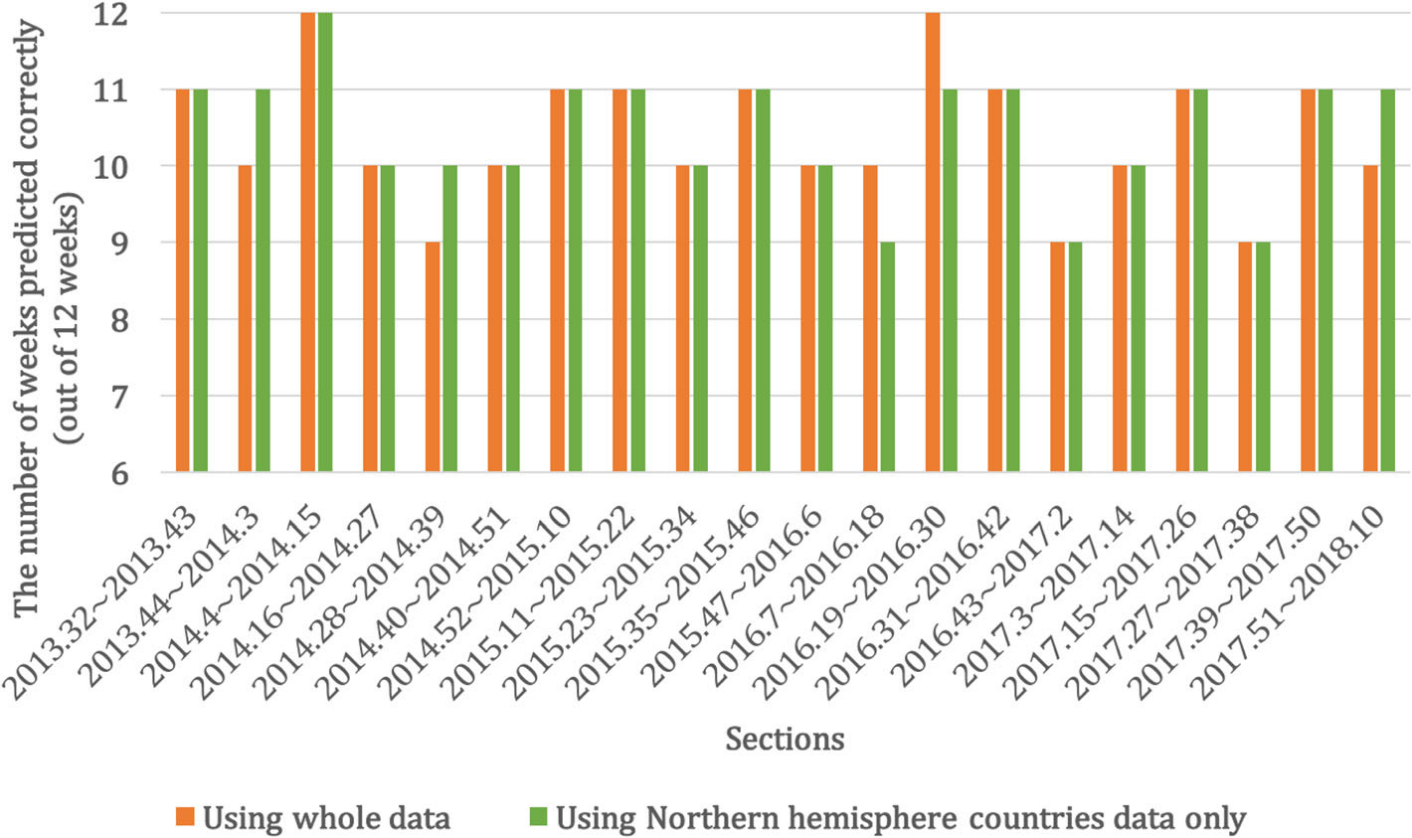
Prediction result of the number of weeks predicted correctly for 20 different sections

Figure 9 shows the accuracy of prediction. The average accuracy for 12 different sections was 0.867 when using all data and 0.871 when using data from Northern Hemisphere countries only, while the minimum was 0.75 and the maximum was 1.0. In Table 4, the accuracy and RMSE of each section and its average are presented. While the accuracy was under 0.8 in the three sections of 2014.28–2014.39, 2016.43–2017.2, and 2017.27–2017.38, the rate of visits of hospitals for ILI was between (0.7 and 1.2), which was not a sharply increasing or decreasing section for the results which used all data. However, every section, except for one section where the rate of patients increased or decreased dramatically, showed more than 0.8 prediction accuracy. The accuracy was slightly higher when using data from Northern Hemisphere countries only than when all data was used.

**Table 4:** Accuracy and RMSE of prediction for the rate of hospital visits for ILI using SVM from the 32nd week of 2013 to the 10th week of 2018

Period	Using all data		Using Northern Hemisphere countries data only	
	Accuracy	RMSE	Accuracy	RMSE
2013.07.30 ~ 2013.10.21	0.917	0.656	0.917	0.298
2013.10.22 ~ 2014.01.13	0.833	1.081	0.917	0.381
2014.01.14 ~ 2014.04.07	1.000	0.365	1.000	0.316
2014.04.08 ~ 2014.06.30	0.833	0.333	0.833	0.296
2014.07.01 ~ 2014.09.22	0.750	0.083	0.833	0.122
2014.09.23 ~ 2014.12.15	0.833	1.258	0.833	0.177
2014.12.16 ~ 2015.03.09	0.917	0.759	0.917	0.694
2015.03.10 ~ 2015.06.01	0.917	0.353	0.917	0.331
2015.06.02 ~ 2015.08.24	0.833	0.238	0.833	0.188
2015.08.25 ~ 2015.11.16	0.917	0.780	0.917	0.295
2015.11.17 ~ 2016.02.08	0.833	2.574	0.833	0.936
2016.02.09 ~ 2016.05.02	0.833	0.273	0.750	1.163
2016.05.03 ~ 2016.07.25	1.000	0.106	0.917	0.132
2016.07.26 ~ 2016.10.17	0.917	0.473	0.917	0.314
2016.10.18 ~ 2017.01.09	0.750	0.178	0.750	0.374
2017.01.10 ~ 2017.04.03	0.833	0.617	0.833	0.463
2017.04.04 ~ 2017.06.26	0.917	0.056	0.917	0.102
2017.06.27 ~ 2017.09.18	0.750	0.187	0.750	0.455
2017.09.19 ~ 2017.12.11	0.917	0.665	0.917	0.575
2017.12.12 ~ 2018.03.05	0.833	1.184	0.917	0.315
Average	0.867	0.611	0.871	0.396

**Fig. 10:**
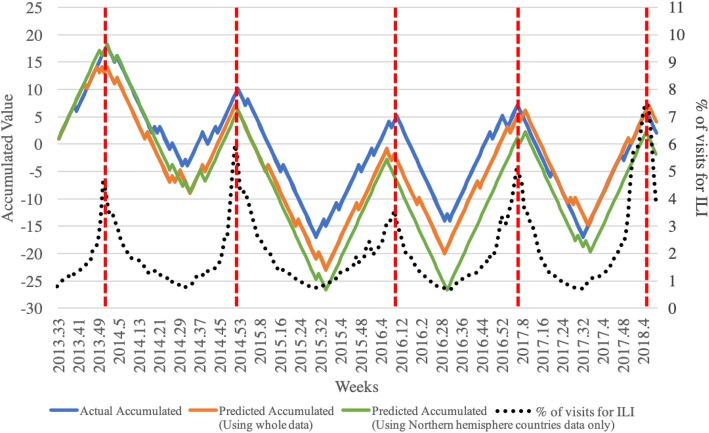
Graph of the cumulative value of the hospital patient visit rate due to the ILI, with the rate increased to ‘+1’, ‘-1’. The black dotted line represents the actual rate of hospital visits for ILI, and uses the right-hand y-axis. The blue, orange, and green lines respectively represent the actual cumulative value of labels and the predicted cumulative value of labels when all data are used and the predicted cumulative value of labels when using data from Northern Hemisphere countries only, using the left-hand y-axis of the graph

Cases where the patient number increased are assigned the label ‘+ 1’ while those where it decreased are assigned the label ‘-1’, and in order to discover when the peak of the number of patients would be with the predicted results, the predicted labels of 240 weeks included in 20 sections are accumulated, as shown in Fig. 10. In the figure, the black dotted line represents the actual rate of hospital visits for ILI, and it uses the right-hand y-axis. The blue, orange, and green lines respectively represent the actual cumulative value of labels, the predicted cumulative value of labels when all data are used, and the predicted cumulative value of labels when only data from Northern Hemisphere countries are used, using the left-hand y-axis of the graph. As shown in red dotted lines in Fig. 10, it allows for the identification of when the peak rate of hospital visits for ILI would be, using the predicted labels.

**Fig. 11:**
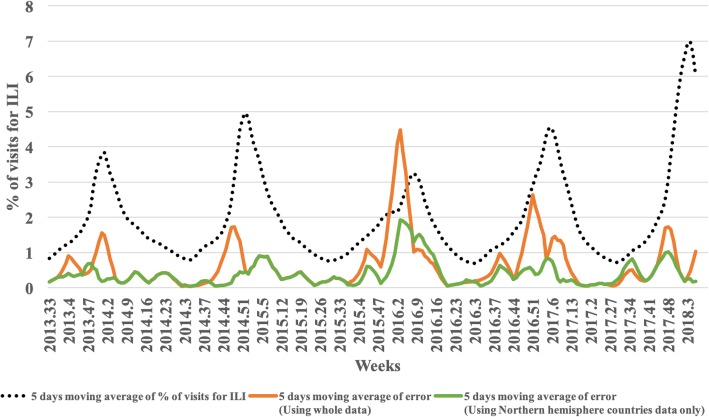
Graph of five-day moving average of ILI patient ratio against the five-day moving average of error between the actual ILI patient ratio and predicted ILI patient ratio

**Fig. 12:**
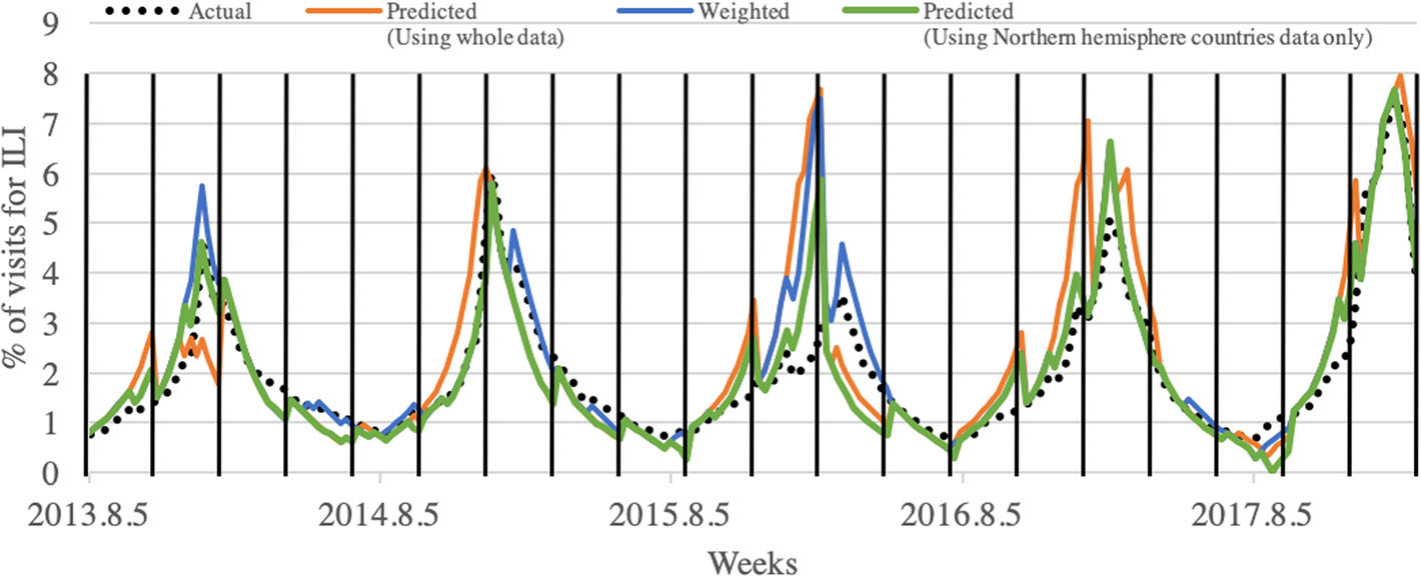
Actual rate of hospital visits for ILI and the estimated rate of the 20 section. The black dotted line represents the actual ILI patient ratio. The blue and orange lines represent the actual value of labels with weighting index applied and the predicted value of labels with weighting index applied, respectively

Using the predicted results and weighting index in Table 2, the rate of hospital visits for ILI is estimated as shown in Fig. 12. As described in Fig. 3, the weighting index was applied to every week where the section starts, using the rate of the previous week, and estimating the rates of 12 weeks. The average RMSE for 20 sections was 0.611 with a minimum of 0.056 and maximum of 2.574 when all data was used, while the average RMSE was 0.396 with a minimum of 0.102 and maximum of 1.163 when only data from Northern Hemisphere countries was used, and the overall error changes throughout the predicted period are shown in Fig. 11. In Fig. 11, the five-day moving average of ILI patient ratio is plotted against the five-day moving average of error between the actual ILI patient ratio and predicted ILI patient ratio. As shown in Fig. 11, out of the 20 divided sections, the second, sixth, seventh, 11th, 12th, 15th, 16th, and 20th sections include the ILI patient ratio peak week of each year. The average RMSEs of these in season sections were 0.991 and 0.563, which could be considered reasonable, even when ILI activity is near maximal as the error was less than 1%. In Fig. 12, the blue line represents the actual rate, while the orange and green lines represent the rates estimated using the weighting index and predicted label, respectively. The results of applying the weighting index to the predicted label follow the same overall trend as the actual value, but quickly increase the estimated ILI patient ratio value by over weighting the value where the value of the graph is increasing rapidly.

## Discussion

**Fig. 13:**
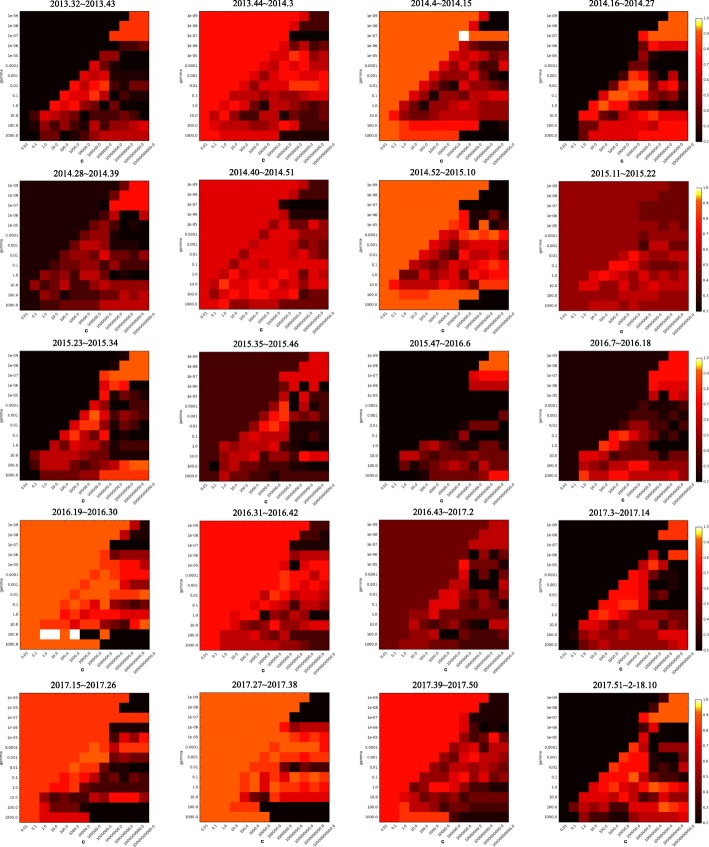
Model performances for 20 different sections with (13 × 13) parameter combinations when all collected data are used

**Fig. 14:**
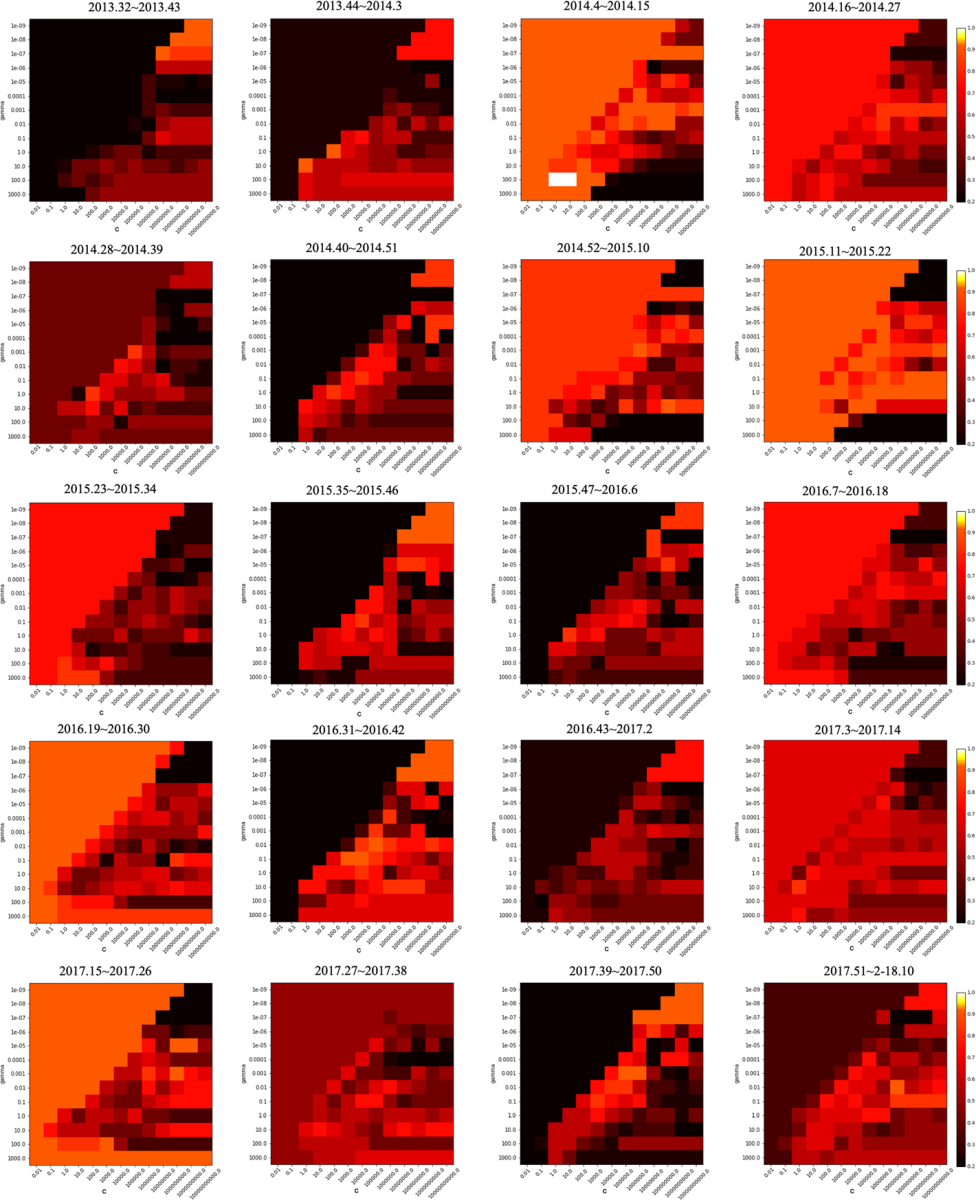
Model performances for 20 different sections with (13 × 13) parameter combinations when only data from Northern Hemisphere countries are used

Although the results of this study show the possibility of using news articles for influenza prediction, there are still several obstacles to resolve. First, the performance of the SVM model is substantially affected by the parameter settings. The smaller the value of parameter C, the more restrictive the model is and the less influential each data point is. In other words, the larger the value of parameter C, the more influential each data point is, and the more it bends the hyperplane. In addition, the smaller the value of *γ*, the greater the radius of the Gaussian kernel, leading many points to be considered closer. The performances of the parameter combinations of 13 × 13 used in this study vary as shown in Figs. 13 and 14. The 20 heat maps shown represent the performance of SVM for 20 different sections. The brighter the red color, the more accurate it is, while the darker the color, the less accurate it is. While more than half of the area is marked with bright red, there is still a dark side. As wrong parameter settings can lead to poor model performance, the results may vary dramatically. Thus, further works, such as research into a method that is less affected by the parameter settings, or a method that rapidly identifies the optimal parameter settings, are required.

Second, even though the model is able to approximately estimate the rate of hospital visits for ILI by applying the suggested weighting index, large gaps exist between the real value and estimated value in certain sections. The annual number of influenza patients increases or decreases due to several reasons, such as climatic status; thus, the weighting index should be fixed annually, reflecting some of the features that affect influenza spread. As the weighting index that is suggested in this study is very basic and uses only an average value based on past data, more research is required for a flexible weighting index.

Third, as shown in Fig. 11 and Table 4, the difference in mean accuracy is not substantial, but the RMSE showed a significant difference. This can be interpreted as indicating that the predictive accuracy at important points was better when using data from countries in the Northern Hemisphere when predicting the change of ILI patient ratio using news article data. In order to verify if there are advantages to using data from countries which are highly correlated with particular infectious disease, additional studies predicting not only the ILI patient ratio but also the patient ratio of some other infectious diseases, such as dengue or Middle East respiratory syndrome, are required.

Finally, the model proposed in this research makes predictions 1 week after the data that has been used to train. The proposed method can predict the number of influenza patients about two to 3 weeks later, because it usually takes about one to 2 weeks to aggregate the number of influenza patients. However, the further we predict the future, the more useful the results will be. Thus, a method that can predict more than two to 3 weeks ahead needs to be studied.

## Conclusion

In this study, we propose using news article data with SVM to estimate the number of influenza patients. The proposed method is advantageous in terms of collecting data, as news articles are easily collectable through the Internet service, and using this accumulated data, it is possible to predict if the number of patients will increase or decrease. Furthermore, with the predicted labels, the actual rate of hospital visits for ILI can be estimated. News article data is usually easier to access than clinical data generated from hospital or climatic data, and is immediately available to be accessed. The average accuracy for predicting the increase or decrease of the number of ILI patients for 20 sections composed of 12 weeks was 0.867 and 0.871, respectively, and the RMSE for 20 sections using the weighting index showed 0.611 and 0.396, respectively. Figure 10 shows that it is possible to discover if the number of patients will increase or decrease using only the results of variance prediction. However, if the peak is discovered using only the result of variance, it is not clear if the peak is a global or local peak. For this reason, we suggest using the weighting index; thus, it is possible to identify if the peak is global or local and also to estimate how high it is. The weighting index controls the intensity of increasing rate and/or decreasing rate when estimating the future ILI patient ratio. Thus, it is available to see when the ILI patient ratio dramatically increases and/or decreases and identify if it is the global or local peak. In addition, we plan to carry out more studies on discovering a new method with which to define objective weights for each ILI patient ratio level. Accumulating the clinical and climatic data which are used as core materials for conventional influenza prediction research is not easy. Thus, in order to remedy the problems of conventional data, in this research, features related to influenza spread are extracted from news articles provided by Internet service, and are used to predict whether the number of hospital visits due to ILI will increase or decrease.

## Abbreviations

CHP: Centre for health protection
ILI: Influenza-like illness
NLP: Natural Language Processing
SVM: Support vector machine
TI: Technical indicators
CBOW: Continuous bag-of-words
RMSE: Root mean square error
